# Facilitators and barriers to participation and scale-up of a non-specialist delivered psychological intervention for adolescents in low-resourced settings: a process evaluation

**DOI:** 10.1186/s12889-025-21914-1

**Published:** 2025-02-21

**Authors:** Alissa M. Terp, Rand Habashneh, Felicity L. Brown, Adnan Abualhaija, Ibrahim S. Aqel, Maha Ghatasheh, Richard Bryant, Mark J. D. Jordans, Aiysha Malik, Ellenor Mittendorfer-Rutz, Aemal Akhtar

**Affiliations:** 1 School of Psychology, University of New South Wales, Sydney, Australia; 2 Institute for Family Health, King Hussein Foundation, Amman, Jordan; 3 Research and Development Department, War Child Alliance, Amsterdam, The Netherlands; 4 Amsterdam Institute of Social Science Research, University of Amsterdam, Amsterdam, the Netherlands; 5 Department of Mental Health, Brain Health and Substance Use, World Health Organization, Geneva, Switzerland; 6 Department of Clinical Neuroscience, Division of Insurance Medicine, Karolinska Institutet, Stockholm, Sweden

**Keywords:** Psychological intervention, Mental health, Refugees, Adolescents, Low- and middle-income countries, Humanitarian emergencies

## Abstract

Globally, the number of refugees and displaced individuals has surpassed 100 million for the first time in history. Refugees are more likely than non-refugee populations to experience psychological distress and develop mental disorders. Early Adolescent Skills for Emotion (EASE), developed by the World Health Organization, is a potentially scalable task-sharing intervention targeting symptoms of internalizing disorders such as depression and anxiety for 10–15 years old and their caregiver. Prior to this study a randomized controlled trial in Amman, Jordan was conducted showing effectiveness of EASE reducing caregiver distress and inconsistent disciplinary parenting as well as reducing internalizing problems in adolescence. This study aims to explore individual and contextual barriers and facilitators for scaling the EASE intervention for Syrian refugees in Jordan. Ten semi-structured interviews and four focus group discussions were conducted between October 2020 and February 2023 with five key mental health and psychosocial support (MHPSS) informants, eight EASE providers, 11 adolescents, and 12 caregivers. Purposeful quota sampling technique was used to recruit participants with attention to sex and age and number of interviews and focus group discussions determined through empirical saturation. Inductive and deductive codes were utilized in a six-step thematic analysis. Participants reported a beneficial impact of EASE sessions in terms of reducing experienced anxiety levels, improved communication between adolescents and caregivers, reduced feelings of anger and jealousy, and improved familial relationships. Individual participation was hampered by transportation issues, scheduling conflicts, gender-mixed groups, and competing responsibilities. Scale-up facilitators included; increased mental health awareness, perceived low cost of EASE, feasibility of delivery by non-specialists, and an intervention engaging both adolescents and caregivers. Barriers included location, online sessions, sustainability, general implementation issues caused by individual barriers, and some concerns about non-specialists. Results add nuances not detected in the previous randomized control trial in Jordan and provide important context for understanding effectiveness results. Future research should investigate the cost-effectiveness of EASE along with stepped-care implementation models to provide EASE within existing health systems.

## Introduction

In 2022, the number of persons forcibly displaced due to humanitarian crises surpassed 100 million for the first time [1]. The countries hosting the majority of refugees are bordering countries, often low- and middle-income countries (LMICs) with already strained healthcare systems, often resulting in limited availability of services for displaced populations [2]. These populations are at heightened risk of experiencing psychological distress and further developing mental disorders compared to non-refugee populations [3]. It is estimated that, on average, 20% of people living in conflict-affected environments have a mental disorder [4] (e.g., anxiety and depression). Mental disorders account for 13% of the global disease burden for children and adolescents [5]. Children and adolescents who have witnessed conflict and humanitarian emergencies have higher rates of mental health issues [6]. In LMICs, mental health services are largely aimed at adult populations, with few child and adolescent services leading to many children and adolescents not receiving the mental health care they need [7].

To address this vast mental health treatment gap [8] facing younger populations, scalable psychological interventions may be helpful in expanding potential for treatment options and increasing accessibility. Scalability is defined as “the ability of a health intervention shown to be efficacious on a small scale or under controlled conditions to be expanded under real world conditions to reach a greater proportion of the eligible population, while retaining effectiveness” [9]. There is increasing evidence of the feasibility and effectiveness of scalable psychological interventions [10] which adapt evidence-based treatments for delivery by trained and supervised non-specialist providers in a ‘task-sharing’ model, thereby decreasing the number of individuals needing specialised care and reducing the treatment gap [11]. However, numerous challenges with scaling-up psychological interventions in humanitarian contexts have been noted, including: interventions focusing on a single diagnosis and therefore requiring clinical decision making, highly trained staff, and intensive resources [12–14]. These findings led to the development of several low-intensity and transdiagnostic interventions delivered by non-specialists e.g. (Group) Problem Management Plus (PM+) [15, 16], which have shown promise among adults [17–20]. PM + is a low-intensity, evidence-based intervention designed for adults experiencing common mental health problems such as depression and anxiety [16]. Utilizing a transdiagnostic approach, PM + integrates problem-solving and behavioural techniques, aiming to enhance emotional management in five weekly sessions delivered by trained non-specialists [16]. Despite these advances, there remains limited evidence for effective scalable interventions for children and adolescents in LMICs [21] and little understanding of specific treatment strategies that lead to positive outcomes for conflict-affected young people [22]. Previous modified psychological interventions used in humanitarian contexts have been shown to be challenging to scale-up due to focusing on a single mental health conditions, requirement of highly trained staff, and intensive resource requirements [12–14]. These findings led to the development of low-intensity and transdiagnostic interventions delivered by non-specialists under supervision after training [23], such as Early Adolescent Skills for Emotiones (EASE) which, renders it suitable for potential scaleup.

To address this paucity in available interventions for a younger age group the World Health Organization (WHO) developed the EASEMeta intervention, which consists of evidence-informed treatment components, designed to alleviate symptoms of internalizing disorders, such as anxiety and depression, in young adolescents aged 10–15 years [24]. Session themes and focuses of EASE sessions are showed in Table 1.

**Table 1:** Session themes and focus in EASE for adolescents and caregivers

Themes and focus		
Session	Adolescents	Caregivers
1	**Understanding My Feelings** Identifying emotions and intends to develop a sense of group feeling	**Psychoeducation and Skills** Emotion identification, slow breathing, active listening
2	**Calming My Body** Taught strategies to calm their emotions when experiencing anger, stress, or anxiety, e.g., diaphragmatic breathing	**Positive Parenting Strategies** Boosting adolescent’s confidence, praise, ending physical discipline
3	**Changing My Actions** Encouraged to change their actions and participate in meaningful	**Caregiver Self-care** Nutrition, sleep, stress reduction
4	**Changing My Actions**	
5	**Managing My Problems** Development of problem-solving skills	
6	**Managing My Problems** Prevention and how to practically implement the strategies taught	
7	**Relapse Prevention**	

The feasibility of implementing EASE has been explored in pilot randomized control trials (RCTs) in refugee communities in Jordan, Lebanon, Tanzania, and schools in Pakistan [25–28]. Studies showed EASE to be feasible and acceptable, however researchers identified areas for improvement such as logistics and caregiver engagement [25–28]. Subsequent to these feasibility studies, effectiveness RCTs were conducted in Jordan [29], Lebanon [30] and Pakistan [31]. Quantitative effectiveness results with urban-living Syrian refugees in Jordan indicated that EASE participants experienced greater reductions of internalizing problems compared to enhanced usual care and a reduction in caregivers’ experiences of psychological distress and inconsistent disciplinary parenting.

Given the positive findings of effectiveness of the intervention in Jordan, there is a need to explore the scalability of the intervention to determine factors that will enhance equitable impact at scale [32]. A recently published process evaluation conducted in North and Akkar governorates of Lebanon following the implementation of EASE identified multiple factors potentially influencing adherence, engagement and perceived impact [33]. Respondents reported positive experiences with the EASE intervention, highlighting notable improvements in adolescent behavior, emotional regulation, and caregiver-child relationships, as well as enhancements in caregivers’ well-being [33]. Nevertheless, EASE’s effectiveness was constrained by families prioritizing basic needs and income-generating activities over mental health, compounded by logistical challenges and stigma associated with mental health interventions [33]. The use of non-specialists proved to be effective, although challenges such as inconsistent working hours and potential burnout were identified, underscoring the necessity for regular supervision and additional training [33]. For the successful scaling up in Lebanon, stakeholders emphasized the importance of integrating the program into existing health and educational structures, while also recognizing that financial constraints and the need for robust quality assurance pose significant challenges [33].

This paper contributes to understanding key factors influencing the scalability of EASE in diverse contexts, by presenting a process evaluation conducted in Jordan following the recently conducted RCT [29]. We aim to specifically explore individual and contextual barriers and facilitators for implementing and scaling the EASE intervention in Jordan, an upper-middle-income country facing a prolonged humanitarian crisis.

## Methodology

### Design

In line with the UK Medical Research Council framework [34, 35] for designing and evaluating complex interventions, this qualitative process evaluation was conducted following a large-scale RCT conducted between June 2019 and September 2020 exploring the effectiveness of EASE (previously published) [29] to explore participant experiences with the intervention and its potential scalability. The Institute for Family Health conducted ten interviews and four focus-group discussions (FGDs) with EASE participants, EASE providers, and key mental health and psychosocial support (MHPSS) informants between October 2020 and February 2023. The WHO Ethical Review Committee (#ERC.0003012) and the Research Ethics Committee of Al Basheer Hospital in Amman, Jordan, (MOH REC1800170) approved the overall EASE research study. Additionally, the Ministry of Planning and International Cooperation in Jordan (# 5/2/10/10818/72) granted permission to conduct this work.

During the RCT, EASE sessions were conducted throughout Amman, Jordan, and participants were provided transportation allowances to maintain participation. The intended implementation was modified during the COVID-19 pandemic which led to online sessions to accommodate changed living conditions during lockdown. Two online sessions were held for approximately 65 adolescents during lockdown but none for caregivers, as all sessions were held prior. Thirty-eight sessions were observed and rated by supervisors and the mean number of EASE sessions attended by adolescents was 4.67 (SD 2.79) and 1.76 (SD 1.22) for caregivers [29]. Prior to implementing the EASE intervention, providers completed eight days of training, developed facilitation skills through role plays, and participated in two supervised practice cycles [29]. Adolescents could participate in seven sessions and caregiver three.

### Setting

Jordan is an upper-middle-income country [36] with over 760,000 registered refugees, where the majority (approximately 83%) live in urban areas outside of refugee camps [37]. The majority are Syrians [37], accounting for 6.6% of Jordan’s population [38], and 27% of the global refugee population prior to the Ukraine crisis [17]. The Jordanian government estimates that the actual number of Syrian refugees is closer to 1.3 million [39]. Prior to the COVID-19 pandemic, around 80% were estimated to live below the national poverty line at 3 USD daily [40]. The ongoing conflict in Syria prevents refugees from returning home [41], and in turn, there is a need to address their heightened rates of mental disorders in host nations. Studies have shown that many Syrian children and adolescent refugees are experiencing psychological problems [42, 43], emphasizing the urgent need for targeted programs. McEwen and colleagues found high levels of depression and anxiety among Syrian refugee children in a study conducted in Lebanon [44] which aligns with the goal of lowering internalizing symptoms such as depression and anxiety symptoms through EASE [24].

### Study procedure and participants

Study data consisted of ten semi-structured interviews and four FGDs with a total study population of 36. Table 2 shows the group, sample size, data collection method, and topics discussed. Interview guides to explore the feasibility, barriers, and facilitators in implementing EASE, focusing on scalability. Topics included barriers and facilitators in EASE recruitment and implementation, health service access, the burden of assessments, and participant satisfaction. The four FGDs consisted of one group of adolescent EASE completers (pre-defined as attending a minimum of five EASE sessions), one group with caregivers who had a child who completed the EASE intervention, and two groups of EASE providers that facilitated adolescent and caregiver sessions.

**Table 2:** Group, sample size, method, and discussed themes

Group	Sample size	Method	Topics
EASE provider (delivery agents in EASE)	(*n* = 8)	FGD (two)	Acceptability and utility of EASE; Fidelity and contamination; Ethics and safety; Recruitment and retention of participants
Completer* (young adolescents in EASE)	(*n* = 6)	FGD (one)	Acceptability; feasibility and utility of the intervention; fidelity and contamination; recruitment and retention; ethics and safety
Completer* (caregivers in EASE)	(*n* = 7)	FGD (one)	Acceptability; feasibility and utility of the intervention; fidelity and contamination; recruitment and retention; ethics and safety
Non-completer** (young adolescents and caregivers in EASE)	(*n* = 6)	Interview (three)	Acceptability; feasibility and utility of the intervention; fidelity and contamination; recruitment and retention; ethics and safety
Never-attended (young adolescents and caregivers)	(*n* = 4)	Interview (two)	Acceptability; feasibility and utility of the intervention; fidelity and contamination; recruitment and retention; ethics and safety
Key MHPSS informants***	(*n* = 5)	Interview (five)	Benefits and challenges of integrating and scaling EASE into service provision

*Defined as attending 5–7 sessions

**Defined as attending less than 5 sessions

*** Two non-governmental organization (NGO) managers, one Jordanian Ministry of Health staff, one psychosocial clinical supervisor, and one specialised mental health care provider

FGDs had six to eight participants and lasted one to three hours. Semi-structured interviews included five key MHPSS informants, six EASE non-completers (NC) (three adolescents and three caregivers), and four who never attended (two adolescents and two caregivers). Key MHPSS informants included two non-governmental organization (NGO) managers, one Jordanian Ministry of Health staff, one psychosocial clinical supervisor, and one specialised mental health care provider. The key MHPSS informants were carefully chosen to represent various groups and provide information from varied perspectives on implementation of mental health interventions. Interview durations ranged between 12 and 54 min and were conducted individually or in pairs (caregiver and adolescent). Due to COVID-19 restrictions, phone interviews were conducted for adolescents and caregivers were conducted over the phone. Informed consent to be contacted for this study was obtained during the informed consent procedure of the RCT. A secondary informed consent was obtained prior to the interview process; prior to adolescents providing assent to participating in the interview, caregivers were approached and consent was obtained for their child to participate.

Research assistants collecting the data were fluent in Arabic and English. They had previous monitoring and evaluation experience and familiarity with conducting qualitative interviews, notetaking, and recording. One interviewer led in asking questions whilst the other recorded both in writing and audioMeta. Interviews were conducted in English or Arabic, depending on the interviewees’ preferences. To avoid any investigator bias on the evaluation of the EASE program, several provisions were put in place. This included having independent interviewers who had no prior interaction with the program participants, ensuring that participants did not feel compelled to offer undue praise or withhold criticism of the program. Data were pseudonymized, with only age and gender as identifiable details. To ensure a suitable sample, purposeful quota sampling technique was used to recruit participants with attention to age and sex. To circumvent bias that may have arisen due to purposeful sampling, a list of participants who met the predetermined cutoff criteria for completers and non-completers was compiled. Participants were subsequently randomly selected until the desired sample size was reached. Number of interviews and FGDs was determined through empirical saturation. Table 3 presents a socio-demographic description of the study population.

**Table 3:** Sociodemographic of the study population

Group	Age	Sex
Adolescent completers	10–15	1 female 5 males
Caregiver completers	32–50	7 females
Non-completer adolescents	11–13	1 female 2 males
Non-completer caregivers	28–45	3 females
Never-attended adolescents	13	1 female 1 male
Never-attended caregivers	45	2 females
Facilitators	28–45	6 females 2 males
Key informants	35–50	2 females 3 males

### Analysis

The analysis focused on identifying facilitators and barriers for individuals and for the potential scalability of a low-intensity psychological intervention delivered by non-specialists in urban-refugee settings. Prior to analysis interviews conducted in Arabic were transcribed, anonymized, and subsequently translated into English by bilingual translators with prior psychosocial experience. Following translation, an independent translator assessed the accuracy of the English translations by comparing the transcribed text with the spoken Arabic interviews. English interviews were transcribed directly and anonymized.

Data were analysed using inductive and deductive thematic analysis, guided by Braun and Clarke’s six-phase approach [45], and using NVivo Release 1.7.1 [46]. After two authors (AMT and AA) familiarised themselves with the data, initial open coding was conducted on the first three interviews and FGDs to generate initial inductive codes. Deductive codes were developed in discussion between the two authors and informed by the semi-structured interview and FGD guides to maintain study focus. However, inductive coding ensured capturing all potentially meaningful experiences or points of view that did not fit within the pre-set deductive codes. Inductive and deductive codes were combined into a structured codebook, applied to the entire dataset and adjusted throughout the process. After all transcripts were coded, AMT and AA reviewed and grouped codes, forming preliminary themes. Lastly, themes were carefully checked for coherence and completeness before being illustrated with relevant quotes. Frequent discussions were held between the two coders, and the codebook revised iteratively where required. In addition, to avoid misinterpretation in the transcripts, data checks were conducted with the Jordan team.

## Results

**Fig. 1:**
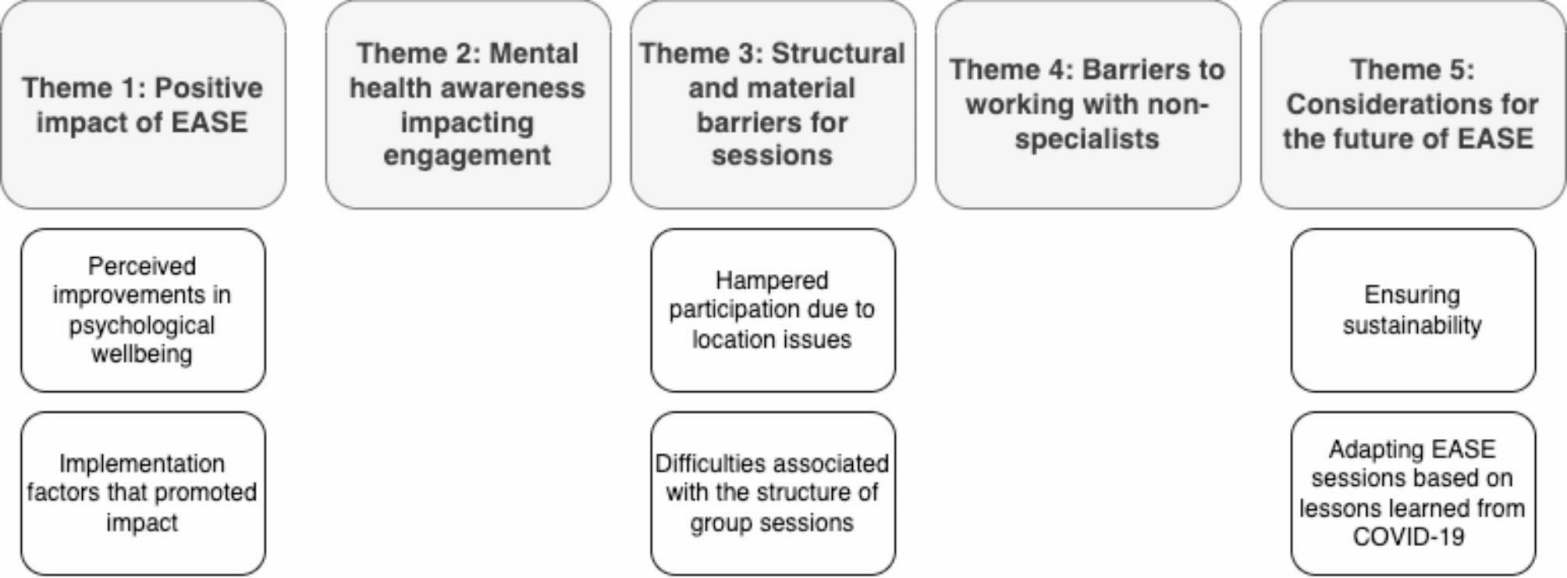
Identified themes and subthemes

The thematic analysis resulted in five themes and six sub-themes, illustrated in Fig. 1.

### Theme 1: positive impact of EASE

#### Perceived improvements in psychological wellbeing

The majority of adolescents and caregivers indicated that taking part in EASE was a positive experience. Caregivers noticed changes in their mood and parenting skills. One caregiver completer (CC) in particular felt an impact on her level of experienced anxiety.

“*In the past*,* I was nervous about my children*,* my husband*,* and my life*,* but after the project*,* I became less nervous ” CC1.*

Adolescents reported positive impacts of the program on their mood, attributing it to various skills taught, particularly a stress management exercise. For instance, some used the technique after arguments with siblings, while another adolescent completer (AC) expressed how this exercise benefited them, stating: *“[.] when I play a sport [and get angry]*,* I walk [away]*,* and it relieves me”*,* AC4.*

EASE had a positive impact on adolescent’s daily lives, fostering improved communication with loved ones. Cited examples included improved communication with a mother through eye contact and one having previously been afraid of punishment when seeking help from caregivers for distress, but following EASE, felt confident to do so. An EASE provider expressed that a lack of positive parenting could lead to struggles for adolescents, however caregiver sessions helped to alleviate this. This was echoed by caregivers:

“*The most important thing is that children want to express to us what is inside them. [.] I now know how to deal with him*,* and I want to know why he is upset or angry*,* what happened to him. I did not deal with him in this way before.“*,* CC2*.

Other beneficial outcomes of participating in EASE expressed by adolescents included: improved friendships, increased understanding and respect for others, learning how to remain calm in heated situations, such as when someone takes their belongings, improved ability to open up, and decreased feelings of jealousy.

*“I noticed that he was changed in terms of nervousness. My son used to get angry about anything*,* and I noticed that he started doing deep breathing exercises and taking a lot of deep breaths”*,* CC4*.

Caregivers observed decreased jealousy, fewer sibling fights, and increased openness as their child shared feelings and daily experiences.

While the taught skills were thought to be helpful and used by most, some adolescents mentioned forgetting to implement them outside of sessions, with one stating being too busy to utilize the skills.

#### Implementation factors that promoted impact

Participants frequently emphasized the positive experience of receiving EASE from non-specialised workers as a key facilitator. Participants overwhelmingly found the session environment comfortable and safe. Caregivers appreciated the guiding approach of EASE providers, fostering an open atmosphere where questions were encouraged. Notably, confidentiality was highly appreciated by caregivers, contributing to a sense of trust. The taught skills were seen as life skills, helping destigmatize mental health for participants, according to key MHPSS informants.

The inclusion of both caregivers and their children allowed participants to cooperate on personal or family issues outside of EASE sessions. According to a key MHPSS informants, EASE was the first intervention that they were made aware of in their work that included both caregivers and adolescents rather than separately. As a result, the intervention was perceived as more efficient because caregivers and adolescents can potentially converse more easily on mental health related topics. The necessity of targeting adolescents was elaborated:

*“The ages here are very important because when you start from 10–14 years old*,* they are like a sponge able to absorb these crises in their way. So here the role of the intervention is to improve their skills and capabilities so that they improve their ability to face problems better.“*,* key MHPSS informants*,* psychosocial clinical supervisor.*

Several logistical aspects of EASE were mentioned as facilitators; a non-complicated, easy-to-follow, concise program that did not consume much time, and not requiring individual family assessment or tailored plans by providers. Key MHPSS informants emphasized the scarcity of mental health professionals in Jordan, highlighting the need for scalable programs like EASE delivered by non-specialists. According to Key MHPSS informants this approach would enable mental health specialists to focus more on severe cases requiring more specialized treatment, such as individual therapy.

As EASE targets psychological states rather than living situations, EASE was perceived to be useful to other people independent of their surroundings or socioeconomic status. All participants in FGDs and interviews agreed on EASE being applicable to other target groups.

*“EASE is based 100% on the psychological state of young people*,* and I am a facilitator who comforts him psychologically. Psychological comfort is different*,* whether poor or rich*,* and the environment from which they came from has nothing to do with the psychological state. We as facilitators may believe that this program seeks to address the difficult feelings of adolescents coming from war and violence through an application that has nothing to do with where the person came from*,* camp or village.“*,* EASE provider 7.*

Regardless of previous experiences or current living conditions, EASE delivering skills applicable throughout life was perceived as beneficial for everyone when learning how to tackle future challenges.

### Theme 2: Mental health awareness impacting engagement

Caregivers perceived psychological support for adolescents and their participation as beneficial both short- and long-term. For instance, it was believed that adolescents’ improved group behaviour as well as enhancing caregivers’ parenting skills would enable adolescents to thrive. The importance of caregiver sessions was reiterated by a number of EASE providers. One facilitator specifically mentioned that *“[…] parents were like a drowning man hanging on to a straw.”*,* EASE provider 2.*

All CCs and one NC caregiver indicated that they communicated openly among their communities on their participation in EASE. Furthermore, they shared what they had been taught at home with other caregivers when they discussed conflicts with their children. This included sharing their own experiences and specific information taught in EASE. This in turn had a snow-ball effect of their family members and peers wishing to participate in EASE. The positive experiences motivated peers to participate, and caregivers wanted their siblings to attend. Additionally, adolescents were all pleased with the sessions, and the majority of both completers and NCs told their friends and family about EASE and some of the adolescents experienced interest from friends and family when telling them. One NCA specifically told friends and relatives about how EASE sessions had improved family relationships.

Key MHPSS informants discussed how the mental health discourse has shifted in Arabic communities, with key MHPSS informants having observed how mental health problems have become more prominent and less stigmatized, and treatment more acceptable.

*“I would say the following is very beautiful. For example Instagram*,* Facebook*,* YouTube*,* make people around the world*,* also the Arabic countries*,* share mental health knowledge.“*,* key MHPSS informants*,* specialised mental health care provider.*

Arabic mental-health professionals have contributed to increased mental health awareness online by participating in anti-stigma campaigns, sharing psychology content, and emphasizing that seeking treatment and vocalizing poor mental health is not wrong. One potential drawback of the encouraging trend indicated by the key MHPSS informants was that Jordan’s limited resources of specialized mental health staff could not meet the increased demand for support, which the growing number of refugees and asylum seekers would only exacerbate; hence the introduction of non-specialised delivered treatments such as EASE in these contexts is potentially timely and relevant.

Key MHPSS informants also indicated that they observed the COVID-19 pandemic helping to destigmatize mental health in Jordan due to shared experiences:

*“[.] such a pandemic is something everybody shares and when you share something with others the stigma is reduced. The ability to talk about it and experience a helpful effect to the problem*,* let’s call the pandemic or mental health issues*,* increases the acceptance of interventions.“*,* key MHPSS informants*,* specialised mental health care provider.*

Experiences from the COVID-19 pandemic enhanced the ability to advocate for scaling-up as people adapt to alternative ways of seeking help (e.g., online sessions and the use of technology).

### Theme 3: structural and material barriers for sessions

#### Hampered participation due to location issues

Effort was taken throughout the trial to ensure EASE sessions were conducted outside of school hours, but session times remained a participation barrier. Additionally, certain sessions overlapped with exam periods, resulting in decreased focus and participation by adolescents. Proposed solutions by participants included weekend or holiday sessions integrated with meals and activities, promoting family interaction.

Among the most significant participation barriers were the physical location of sessions and transportation. Participants received 30 Dinars (~ 42 USD; 3 Dinar/session) to cover for transportation throughout the intervention. Although the locations were set to maximize reachability for participants, based on location proximity to education centres and ease of transportation, interviews revealed that the allowance amount did not entirely cover transportation expenses, resulting in lowered participation. For those that did not require the full use of the transportation allowance, the excess was used on house expenses or for personal use. Another obstacle regarding the location was not all parents being comfortable allowing the adolescents to go to sessions on their own, and both adolescent and caregiver females could not always move freely:

*“As boys*,* it is normal for us to go anywhere*,* but this is more difficult for girls.“*,* AC2*.

Two female adolescents were not allowed to go unaccompanied, with one ceasing participation as no family member could accompany them. Similarly, a female NC caregiver faced obstacles due to childcare responsibilities, preventing her from attending. This concern resonated with many caregivers who had multiple children. Not wanting to take all their children to the centres and difficulty in finding people to care for them resulted in many not attending. A CC specified:

*“My son was a kid*,* and there was no place to leave him*,* and I was afraid to leave him in the house.”*,* CC4.*

EASE providers emphasized that this issue needed a solution for the program to continue and scale-up as it prohibited attendance.

#### Difficulties associated with the structure of group sessions

Initially tense, caregivers and adolescents later found value in group sessions. Caregivers appreciated sharing experiences and advice, while adolescents began assisting each other during tasks. EASE providers noted that when one child opened up, it inspired others to do the same.

*“We felt the positivity of the children at the end of the session*,* the children interacting*,* progressing*,* improving*,* changing*,* and talking about things that we were not able to talk about at the beginning of the session.”*,* EASE provider 2.*

Repeatedly emphasized by EASE providers, gender separation was deemed beneficial. An EASE provider further discussed the specifics during an FGD:

*“[.] I met a father and a guardian with one of his female neighbours. The man was shy because they knew him*,* and he knew them. They just said hello*,* and they go and don’t talk to each other [during the group session].“*, *EASE provider 1.*

EASE providers attributed this discomfort to Syrian customs, hindering cross-gender discussions. One male caregiver stopped attending sessions due to the presence of female participants. A NC caregiver agreed on the benefits of separating genders as she would find a female group more comfortable. Gender issues also impacted materials. Some EASE providers suggested developing age and gender-specific activities and materials due to challenges in approaching adolescents.

*” When you talk to a 14-year-old*,* I feel you are talking to a baby*,* not to a 14-year-old.“*, *EASE providers 1.*

Cooperation and the ability to express oneself, according to EASE providers, varies significantly among the recruited age groups. Furthermore, they believed certain activities and exercises were inappropriate for the entire age group. Despite this, none of the adolescents reported concerns about age difference.

Lastly, EASE providers proposed adjusting session length or reducing group size due to frustrations with limited meaningful interaction, rushed exercises, limiting self-expression, and inhibiting exploration of emotions and experiences within the defined time frame. One modification suggested by some adolescents and EASE providers was the potential benefit of having individual sessions with more time for adolescents as they felt that participants would not feel judged or bullied by others when sharing.

### Theme 4: barriers to working with non-specialists

One key MHPSS informant had positive experiences with non-specialists, finding them to be good-hearted and capable when adequately trained. The key MHPSS informant emphasized their connection to local community and their relatability to participants as crucial for destigmatizing mental health. Despite fruitful experience for participants, multiple key MHPSS informants expressed concerns about non-specialists potentially exceeding the boundaries, as one key MHPSS informant elaborated:

*“According to my experience in dealing with people in Jordan*,* they have a passion in dealing with psychology in order to save their personal lives in the first place*,* but the only fear is that the non-professional service provider may feel an increase in confidence*,* so it increases the scope of their interventions and gives his/her personal opinion on everything*,* and that may lead to negative results.“*, *key MHPSS informants*,* ministry of health staff.*

A key MHPSS informant had experience from other countries where highly motivated and qualified non-specialists, exceeded their role by providing therapeutic or religious counselling. Regardless, it was emphasized that identifying these tendencies during training or supervision is feasible. Nonetheless, non-specialists need to recognize their boundaries and refer special- or severe cases beyond the scope of EASE.

*“[.] they [non-specialists] should understand they are not psychologists and not counsellors because we don’t want them to exceed the limit. They need to know where to stop and [when] to refer the cases to others*,* I think it’s the dilemma in this intervention and the relation between both specialized and nonspecialized it’s strongly recommended to be systemized.”*,* key MHPSS informants*,* NGO manager.*

This concern relatively aligns with the EASE providers feeling restricted by the material and supervisors, hindering their ability to influence activities and teaching approaches for unique session delivery.

*“The content of the manual explicitly constrains us that we must read from within the course [.]. This thing makes the facilitator tense. So I want to tell the child that I have no problem with the sequence he/she requested*,* but if I read this a little bit*,* it makes me feel that I don’t want to read.“*,* EASE providers 6.*

As highlighted, effective collaboration with specialists is essential. A key MHPSS informant (NGO manager) had experienced psychologists and mental health program managers who felt threatened professionally by non-specialists delivering services. This could have a detrimental impact on the collaboration as they did not find non-specialists suitable for the tasks.

Both EASE providers and key MHPSS informants highlighted the necessity of clinical supervision and emphasized the need for more as they felt that EASE providers new to the field should not feel entirely responsible for participants. Despite lengthy wait times, EASE providers expressed satisfaction with supervisors but requested more appropriate planned supervision. EASE providers reported experiencing delays in receiving supervision following the completion of EASE sessions, since these were scheduled weekly. Additionally, co-facilitators of the same EASE groups often had difficulties organizing supervision sessions due to conflicting schedules of their non-EASE responsibilities. Follow-up training which includes a focus on reviewing competencies was a crucial consideration suggested by key MHPSS informants.

### Theme 5: considerations for the future of EASE

#### Ensuring sustainability

Key MHPSS informants and EASE providers mentioned various resource requirements that would need to be considered to ensure sustainability in the future, including materials, location, human resources, and funding. The program was perceived to be low cost due to the comparably small number of sessions per family, non-specialists delivering sessions, and the low number of specialized supervisors. EASE providers identified work opportunities as motivating elements for EASE providers to continue with the program. Sustainable funding sources were deemed necessary for ongoing material production and securing long-term premises.

It was perceived as essential to find sustainable collaborative implementation actors or partners to allow for scale-up. Key MHPSS informants suggested collaboration with the Ministry of Health, Ministry of Social and Development, Ministry of Youth, other NGOs and international NGOs, universities, schools, and the primary health care sector. Schools were emphasized for their consistent location, potential for easily recruitable EASE providers (e.g. trained teachers) and easier access to the target group.

Key MHPSS informants agreed on the importance of collaborating with ministries in Jordan and other contexts where EASE may be implemented to ensure sustainability, although perspectives differed on the timing of involvement. Some suggested initiating at the governmental level, working with centres, while others recommended engaging with other NGOs before involving the government.

*[.] we can’t start on a very big scale to target all the last entities [.] but if we start from the non-governmental organization to build some kind of memorandum of understanding with the public sector then with other ministers*,* that will be possible.”*,* key MHPSS informants*,* psychosocial clinical supervisor.*

Collaborating with various NGOs providing psychosocial interventions, particularly those for 14-17-year-olds, was suggested as potential partnerships. EASE could serve as an initial step, enabling continued enrolment in programs and a deeper understanding of mental health and preventive methods for older adolescents. Emphasis was also placed on identifying sites where refugees felt accepted for effective implementation.

*“[.] if you are refugee in a country*,* going to the same institutions as the local community can be helpful for integration*,* but sometimes there’s also issues of group dynamics. It’s like sometimes it’s helpful to go somewhere*,* which is specific for refugees [.] For example [where] all refugees are more accepted*,* and they are not in competition for the health care services. Then you might integrate it to the local healthcare services if there are issues of power dynamics and jealousy.“*,* key MHPSS informants*,* specialised mental health care provider.*

This adds to another point about incorporating EASE into the health system or collaborating with ministries for targeting a wider audience. It was argued that this would benefit Jordanians as many refugees do not seek assistance from government-led services due to a lack of insurance, social security numbers, and related legislative issues.

Universities were identified as suitable recruitment spot for staff utilizing internships and raising awareness of low-intensity programs. Finally, expanding EASE awareness among family doctors, social workers, clinics, and primary health care providers was suggested to expand its reach.

#### Adapting EASE sessions based on lessons learned from COVID-19

Several COVID-19 related topics were brought up in the five themes. As with many programs implemented during the COVID-19 pandemic, experiences of participating in EASE was altered. Specifically related to theme 1, the pandemic worsened adolescent achievements, regardless of the previous positive impact and multiple adolescents stopped practicing the taught exercises, affecting EASE’s positive impact.

Regarding theme 3, structural and material barriers for sessions, the move to online sessions was supported by key MHPSS informants, aligning with other program implementations during the pandemic. Caregivers were pleased that adolescents could attend EASE online to allow for the completion of the program but participants faced numerous challenges including lack of interaction, privacy, devices, and internet issues despite the distribution of internet data for participants. EASE providers were challenged in managing adolescents online due to lack of training. Running outside to obtain a good connection and having relatives inside the household made it difficult for children to open up and focus. People had difficulty accessing internet platforms, making it even more difficult for participants to concentrate. For CCs, physical sessions with adolescents were preferred since they naturally facilitated more interaction. Given that online sessions were required, one AC would have preferred if these sessions were held for individuals, similarly, a never-attended caregiver wished for the option of attending physical private home sessions.

Related to theme 4, online supervision was welcomed by EASE providers as a positive modification for future flexibility and addressing post-session supervision challenges. Key MHPSS informants found the increase in use of online platforms during the pandemic supportive in adapting EASE to online sessions in the future.

*“And I can say something about using the online training and using online sessions providing mental health sessions by phone*,* which really creates new opportunity for the community to do virtual activities which will be great if we can be part of this.”*,* key MHPSS informants*,* NGO manager.*

## Discussion

The study aimed to explore individual and contextual barriers and facilitators in scaling the EASE intervention for Syrian refugees in Jordan. Participants reported benefits with particular emphasis on specific taught skills (e.g. stress management techniques), improved relationships, lowered levels of experienced anxiety, and decreased acting out in adolescents. Hindered participation factors included transportation, competing responsibilities, scheduling, and gender-mixed groups. Related to the potential scalability of the program, contextual facilitators included increased mental health awareness, perceived low-cost intervention, involvement of non-specialists, and inclusion of caregivers. Concerns about physical locations, online sessions, and session structure needs careful management during scaling up.

In the Jordan RCT, participants showed greater reduction in internalizing problems compared to those receiving enhanced usual care and caregivers experienced less psychological distress and more consistent disciplinary parenting [29]. This process evaluation reinforced these outcomes emphasizing reduced anger issues, acting out, and improved relationships. Participants indicated potential benefit for externalizing problems and caregivers emphasized enhanced positive parenting and participation, stressing the importance of dedicating time to the adolescents and utilizing praise.

Previously, process evaluations focused on internal RCT elements [32]. However, there has been a recent shift towards emphasizing the importance of understanding complex contextual factors potentially influencing the effectiveness and scale-up [32]. Before scaling up interventions based on promising results from existing contexts, it is crucial to evaluate them in new and different settings [32]. This requires process evaluations that can capture the complex contextual elements that may influence effectiveness, feasibility, and reach [32].

Several facilitators aligned with a prior process evaluation of EASE in Lebanon [33], particularly related to improvements in adolescent behaviour, stress management, and self-expression. However, there were differences in implementation and scalability barriers between the two sites. In Lebanon, adolescents' work was a participation barrier, whereas in Jordan, exam periods and school were major challenges. Both process evaluations highlighted physical locations barriers for EASE sessions, but the reasons varied; in Lebanon, buses made transit more convenient, though longer. In Jordan, it was not always possible to cover the total cost of transportation given the urban planning of certain communities in which the program was implemented. Unfortunately, no pickup services were provided for implemented programs which led to participation barriers. A future solution to circumvent these barriers would be to provide transportation for similar programs and to minimize the planning and financial burden for participants.

Stigma surrounding mental health was mentioned as a barrier in Lebanon, but not in Jordan. Previous research in Jordan reported stigma as a significant barrier to mental health care-seeking among Syrian refugees [47], however the study differed as participants were adults selected across Jordan while those in this study had undergone EASE, potentially minimizing the perceived stigma. The main content of EASE appears to be acceptable and efficacious to both participants and key MHPSS informants, however it should be emphasized that EASE may need to be flexible and adaptive to the specific needs of the context in which it is implemented.

EASE in Lebanon and Jordan exemplifies adapting the same intervention to distinct contextual constraints. Unlike in Jordan, stakeholders in Lebanon expressed no concern in working with non-specialists. Prior research indicates that non-specialists can provide effective treatment for anxiety and depression [11]. In Jordan, EASE participants perceived providers as competent, while key MHPSS informants expressed concern about potential role exceeding; key MHPSS informants raised none of these concerns in the PM + study in Jordan [19]. Trust is enhanced when non-specialists share commonalities with participants [20, 48, 49], yet personal familiarity can pose confidentiality challenges [48]. In Jordan, no participation barrier was linked to non-specialists as they were perceived as trustworthy, establishing a safe and inspiring environment. For future scale up it is important to establish a good working relationship and linkage between EASE non-specialists and specialists in the health sector for collaboration on e.g. potential EASE attendants needing a different care.

Both sites emphasized the necessity for additional training and supervision. Weekly group supervision was provided by local trainers, who had been trained in both EASE intervention and supervisory techniques. These local trainers also receive regular supervision from a clinical psychologist, to ensure adherence to the treatment protocol and provide additional support [50]. In the future, supervision should be scheduled with greater consideration for the availability and convenience of EASE providers, ensuring that meetings with both the supervisor and provider occur under appropriate conditions and at mutually suitable times. This is critical as clinical supervision when delivering psychological interventions is a necessity for quality of care. The need for assessing competency in training non-specialist mental health providers has been highlighted previously [51] and has been implemented in other disciplines in LMIC [52]. To address this need, the WHO and UNICEF have established a platform of evidence-based tools and resources for training and assessing provider competencies for psychological care [53], including tailored competency assessment tools specifically for EASE [54]. Adequate clinical supervision has been recognized as crucial in the international MHPSS community, leading to the development of an integrated supervision model by the Red Cross for MHPSS [55, 56]. Collective efforts are underway among both researchers and the NGO community to address these important points allowing for improved implementation of non-specialist delivering psychological interventions in the future and enhancing potential scalability. Regarding EASE in particular, global efforts are currently underway to collect further quantitative and qualitative data on implementation initiatives. This will further enhance our understanding on the effectiveness and user experiences with the intervention and expanding the generalizability of prior and future work across different contexts.

Logistical challenges and competing responsibilities are recurrent challenges in other potentially scalable psychological programs [19, 57–59]. As primary caregivers, women [60], faced participation challenges due to competing responsibilities. Previous evaluations of PM + in Jordan, a transdiagnostic psychological intervention for adults, highlighted that providing childcare during sessions improved female attendance [19]. Changing the venue to a more accessible location or offering need-based transportation allowance could help address transportation challenges and improve participation. Moreover, accommodating personal preferences, such as offering individual and group sessions, and mixed or separated gender groups [19, 20, 61], is essential to overcome identified participation barriers. Individual sessions were mentioned to help adolescents feel less judged when sharing; this is one possible avenue to explore, however, to retain positive aspects of group interactions and to promote the development of broader interpersonal skills, it remains important to also explore ways to prevent any bullying in group interventions. Providing additional training for providers on how to prevent and manage bulling, establishing communication guidelines and expectations with participant groups from the first session, along with fostering a sense of community and encouraging connects among adolescents, may contribute to effective bullying prevention in the context of implementing EASE.

Schools were mentioned as an appropriate scale-up setting and have been used previously in LMIC settings as improved mental health can potentially positively affect school attendance and increase academic skills [62, 63]. Schools have often been thought of as desirable locations for these programs as they allow for increased recruitment of children [6, 62], increasing potential early identifications, prevention, and management of mental health issues.

EASE implementation in schools was explored in Pakistan, demonstrating feasibility and acceptability effectively addressing location and transportation barriers for adolescents, although caregivers still faced challenges related to travel and competing responsibilities [28]. Notably, while universal mental health interventions delivered in schools have varied results, with well-controlled effectiveness trials yielding non-significant results [62], considering alternatives to reach children who do not attend schools remains imperative in LMICs.

Key MHPSS informants emphasized the necessity of involving ministries in the scale-up process to maintain sustainability, aligning with previous critical scale-up factors identified; a sentiment which aligns with prior research [17] and best practices to strengthen and support government-lead systems rather than developing parallel systems [64]. Planned strengthening of health-care and community-based support for refugees are one of the focus areas of the Jordanian *National Mental Health and Substance Use Action Plan for 2022–*2026 [65]. Furthermore, the Jordanian Ministry of Health aims to improve mental health literacy and reduce mental health stigma and discrimination [65]. Increased promotion of improved mental health policy and the initiatives outlined in the current mental health action plan could work towards foster a more natural integration and adherence to scalable psychological interventions such as EASE which are currently being used by NGOs to support refugees in Jordan. Additionally, integration of these programs can contribute to alleviating the burden on the healthcare system in Jordan, which has a scarcity of mental health professionals [38].

Collaborations between the educational and health sectors have already been found to improve access to youth services in low-resource settings [28] supporting one of three potential identified solutions for ensuring sustainability in PM + by the inclusion of university interns in EASE and raised awareness of low-resource psychological interventions [19]. Secondly, partnering with UN agencies and the Jordanian government was considered valuable for sustainable funding and government ownership [19]. However, budgetary issues in Jordan, particularly the lack of mental health funding, may hinder this [38]. This is a widespread challenge to scale-up psychosocial support and mental health interventions in LMICs [17].

Lastly, the change of discourse and focus on mental health, particularly post-COVID-19, was an identified catalyst for scale-up in Jordan. No political challenges were highlighted, mirroring findings from the PM + study in Jordan. Integrating mental health services through NGOs or a combined NGO-government facility was the best recognized approach before full government integration [19]. This could benefit from a stepped-care solution, integrating interventions into existing care systems [20, 66]. NGOs have previously been found to be more successful in reaching populations than the government because they are more invested, motivated, and able to implement programs more rapidly [67]. Funding initiatives like EASE in aid-dependent nations might be challenging, recommending co-funding for improved sustainability from e.g., private, national, and international investors [19]. This option is already in place in Jordan, where the Ministry of Health and NGOs collaborate to fund health clinics [19]. Finally, it was stressed how critical raising awareness of EASE at other services is to reach more people.

### Strengths and limitations

This research has some limitations, including potential loss of input during translation from Arabic to English, addressed by using professional bilingual translators with psychological backgrounds. Additionally, the COVID-19 pandemic led to phone interviews, possibly affecting dialogue flow. Likewise, interviewing adolescents and caregivers simultaneously could potentially have hindered comfort with expressing themselves. Interviews and FGDs were conducted rapidly after the end of the sessions for participants and EASE providers to minimize recall bias. Final interviews were conducted with key MHPSS informants when scale-up initiatives were initiated in Jordan and due to this, there was a significant interval of almost two years between the end of the trial and these interviews. However, it is not expected that this would cause significant bias as key MHPSS informants were not a part of the trial and the interviews focused primarily on potential scalability rather than recollecting EASE experiences.

Finally, the sampling technique, while useful for gathering diverse perspectives, had some limitations. Using purposeful sampling allowed insights from various group respondents, but conducting multiple FGDs within each group might have provided a more in-depth understanding of individual barriers and facilitators. This approach, vulnerable to researcher errors might be incapable of generalizing research findings [68]. Nonetheless, this study successfully attracted various participant groups; completers, NCs, and never attendees, to ensure a range of views were captured.

## Conclusion

The findings have implications for future task-sharing intervention scaling-up in conflict-affected populations that has been displaced to LMICs. To ensure successful implementation, it is essential to eliminate participation barriers including transportation and scheduling by offering flexible session formats (e.g. remote sessions), logistical support, and childcare during sessions. Training and supervising non-specialists is vital in maintaining their competencies and maintaining their own wellbeing. Furthermore, it is important to ensure long-term funding through partnerships with government entities, NGOs and international organizations as well as exploring co-funding options. Involving government ministries from the outset can enhance reach and integration into existing health systems. Future research should focus on testing the feasibility of implementing EASE in the health system potentially as a stepped-care approach and investigating the cost-effectiveness. EASE is currently viewed as a low-cost intervention, but testing the real cost-effectiveness is critical to ensuring government buy-in.

## Data Availability

The data used and analysed during the current study are available from Aemal Akhtar (aemal.akhtar@ki.se) upon reasonable request.

## Abbreviations

AC: Adolescent completer
CC: Caregiver completer
EASE: Early Adolescent Skills for Emotions
FGD: Focus-group discussions
LMIC: Low- and middle-income country
MHPSS: Mental health and psychosocial support
NC: Non-completer
PM+: Problem Management Plus
RCT: Randomized control trial
WHO: World Health Organization
